# The Mechanisms and Biomedical Applications of an NIR BODIPY-Based Switchable Fluorescent Probe

**DOI:** 10.3390/ijms18020384

**Published:** 2017-02-11

**Authors:** Bingbing Cheng, Venugopal Bandi, Shuai Yu, Francis D’Souza, Kytai T. Nguyen, Yi Hong, Liping Tang, Baohong Yuan

**Affiliations:** 1 Ultrasound and Optical Imaging Laboratory, Department of Bioengineering, The University of Texas at Arlington, Arlington, TX 76019, USA; bingbing.cheng@mavs.uta.edu (B.C.); shuai.yu@mavs.uta.edu (S.Y.); 2 Joint Biomedical Engineering Program, The University of Texas at Arlington and The University of Texas Southwestern Medical Center at Dallas, Dallas, TX 75390, USA; knguyen@uta.edu (K.T.N.); yihong@uta.edu (Y.H.); ltang@uta.edu (L.T.); 3 Department of Chemistry, University of North Texas, Denton, TX 76203, USA; venu235@yahoo.com (V.B.); francis.dsouza@unt.edu (F.D.); 4 Department of Bioengineering, The University of Texas at Arlington, Arlington, TX 76019, USA

**Keywords:** aza-BODIPY, environment-sensitive fluorophore, switchable fluorescent probe, near-infrared, wash-free live-cell imaging, in vivo fluorescence imaging, temperature sensing, USF imaging

## Abstract

Highly environment-sensitive fluorophores have been desired for many biomedical applications. Because of the noninvasive operation, high sensitivity, and high specificity to the microenvironment change, they can be used as excellent probes for fluorescence sensing/imaging, cell tracking/imaging, molecular imaging for cancer, and so on (i.e., polarity, viscosity, temperature, or pH measurement). In this work, investigations of the switching mechanism of a recently reported near-infrared environment-sensitive fluorophore, ADP(CA)_2_, were conducted. Besides, multiple potential biomedical applications of this switchable fluorescent probe have been demonstrated, including wash-free live-cell fluorescence imaging, in vivo tissue fluorescence imaging, temperature sensing, and ultrasound-switchable fluorescence (USF) imaging. The fluorescence of the ADP(CA)_2_ is extremely sensitive to the microenvironment, especially polarity and viscosity. Our investigations showed that the fluorescence of ADP(CA)_2_ can be switched on by low polarity, high viscosity, or the presence of protein and surfactants. In wash-free live-cell imaging, the fluorescence of ADP(CA)_2_ inside cells was found much brighter than the dye-containing medium and was retained for at least two days. In all of the fluorescence imaging applications conducted in this study, high target-to-noise (>5-fold) was achieved. In addition, a high temperature sensitivity (73-fold per Celsius degree) of ADP(CA)_2_-based temperature probes was found in temperature sensing.

## 1. Introduction

Fluorescence imaging and sensing in cells or tissues gain great interest due to the unique features, such as non-physical-contact operation, high sensitivity and specificity, unique fluorescence spectrum and lifetime, etc. [1,2,3,4,5,6,7]. Recently, switchable fluorescent probes (SFPs) have been attracting much attention for imaging-specific environmental changes and molecular targets. This is because: (i) SFP can provide high target-to-background ratio [3,8] and therefore has high detection sensitivity; and (ii) SFP can specifically respond to certain stimuli and therefore has high specificity. Generally, the fluorescence of SFP is undetectable or very weak and therefore it generates a dark or low background. The probes can be switched on by a specific stimulus, such as a physical (temperature, polarity, viscosity, etc.), chemical (pH, ion concentration, etc.) or biological (interaction with biomolecules, proteins or DNA, etc.) stimulation. 

Many types of SFPs have been developed during the past years. One of the commonly used SFPs is based on environment-sensitive fluorescent dyes. For example, a molecular rotor is a fluorescent molecule that can undergo an intra-molecular twisting motion in the excited state. This twisted excitation state (in some molecular rotors) can lead to significant non-radiation relaxation. Thus, the quantum yield may be extremely low if the formation rate of the twisted state is high. However, when the local micro-viscosity increases, the rotation capability of the molecular rotors is restricted or disabled, and the non-radiation relaxation is suppressed. The quantum yield can dramatically increase and then the molecules are switched on by the viscosity change [9,10,11,12,13,14]. Binding with proteins or cell membrane or other cell organelles can lead to the increase of molecular viscosity or the restriction of the rotation, and then switch on the fluorescence too. Thus, some molecular rotors can be used for wash-free imaging of proteins or cells because the signal from those unbound molecules is ignorable.

In addition to directly using fluorescent molecules, environment-sensitive-dye-conjugated polymers or nanoparticles are also developed as SFPs for different applications. For example, polarity- and viscosity-sensitive fluorescent dyes have been conjugated on (or encapsulated into) a temperature-sensitive polymer (or nanoparticle) [15,16,17,18,19]. When the temperature of the polymer (or nanoparticle) crosses around a threshold (LCST: the lower critical solution temperature), the polymer (or nanoparticle) experiences a transition between a hydrophilic and a hydrophobic phase. This phase transition leads to the dye switches between a water-rich and a polymer-rich microenvironment. When the temperature is below the LCST, the dye fluoresces weakly in the water-rich microenvironments because water provides a high polar and nonviscous microenvironment that increases the rate of the non-radiation relaxation of the excited fluorophores. In contrast, when the temperature is above the LCST, the dye fluoresces strongly in the polymer-rich microenvironment because the polymer provides a relatively low polar and viscous microenvironment, which can suppress the nonradiative decay rate of the excited fluorophores [20,21]. This type of fluorescence switching is reversible and can be used for temperature imaging in cells or tissues.

Besides the temperature imaging, based on the same type of SFPs, we recently developed a new imaging technique, ultrasound-switchable fluorescence (USF), for visualizing tissue microstructures (such as microvessels) in centimeter-deep tissues. The basic idea of USF is to use a focused ultrasound wave to externally increase the tissue temperature at the focus slightly above the LCST and then switch on the fluorescence of the probes. With the help of environment-sensitive dye-encapsulated SFPs, it has been found that USF can provide high-resolution fluorescence images in deep tissue, which has not been achieved by other optical imaging methods [16,17,19,22].

While many different SFPs are being continuously discovered and developed, and their applications are also being intensively investigated [23,24], one of the most important parameters to quantify a SFP is the switching ratio of the fluorescence intensity between the ON and OFF states (I_ON_/I_OFF_). A high ratio is preferred for achieving a good signal-to-noise ratio (SNR). In addition, a SFP that only responds to a specific or certain environment stimuli is preferred for achieving high specificity. For applications in cells or tissues, near infrared (NIR) excitation and emission wavelengths are also needed for achieving a large penetration depth (in tissue) and low cell or tissue autofluorescence.

In this study, we investigated the switching mechanism of a recently reported NIR aza-BODPIY-based fluorescent dye (ADP(CA)_2_) [19] and its potential applications as a SFP in different formats (dye itself and dye-conjugated polymers), including wash-free cell imaging, in vivo tissue imaging, temperature sensing, and tissue USF imaging. In the previous report, it has been found that this dye is extremely and uniquely sensitive to the polarity and viscosity of the surrounding environment, but not physiological relevant pH or ion concentration [19]. Further mechanism investigations were reported in this work. We concluded that ADP(CA)_2_ is an excellent candidate for SFP applications because of its high ratio of I_ON_/I_OFF_ and high specificity to environmental polarity and viscosity. In addition, its peak excitation and emission wavelengths are located at 683 and 717 nm, respectively, which makes it suitable for cell and tissue studies. Lastly, this dye has two carboxyl conjugation groups and can be used for conjugation with other units via amine groups (such as temperature sensitive polymers in this study). In summary, this dye is an excellent candidate for wash-free live-cell imaging, in vivo fluorescence imaging, temperature sensing, tissue USF imaging, and other potential SFP applications.

## 2. Results and Discussion

### 2.1. Switching Mechanisms of ADP(CA)_2_

**Figure 1:**
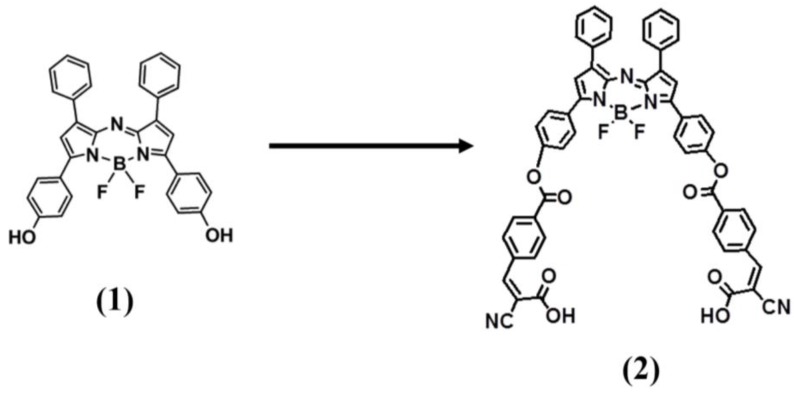
Chemical Structures and synthesis routes of ADP(CA)_2_.

The synthesis of ADP(CA)_2_ (Compound (**2**)) has been introduced in our previous publication [19]. It shows an absorption peak at ~683 nm in dichloromethane (DCM), while its fluorescence emission peak is located at ~717 nm. No significant difference in the fluorescence emission wavelength is found between the reagent (Compound (**1**) [25]) and the product (ADP(CA)_2_). Both of the chemical structures are shown in Figure 1. This result indicates that the functionalization of two cyanocinnamic acid (CA) groups does not affect the Stokes’ shift.

#### 2.1.1. Fluorescence Response to Polarity 

**Figure 2:**
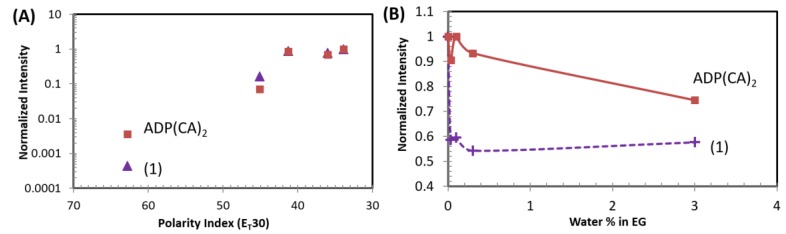
(**A**) Polarity-dependent fluorescence intensity of ADP(CA)_2_ and Compound (**1**); Five solvents with different polarity index [28] were employed, which are, from left to right, water (62.8), dimethyl sulfoxide (DMSO, 45.1), 1,2-dichloroethane (41.3), 1,4-dioxane (36) and toluene (33.9). (**B**) Fluorescence intensity of ADP(CA)_2_ and Compound (**1**) in a water/ethylene glycol mixture with different compositions; Excitation: 655 nm; Emission filter: 711/25 band-pass; Laser energy: 50 pJ (**A**) and 140 pJ (**B**); EG: ethylene glycol.

Our results indicate that ADP(CA)_2_ is a solvatochromic fluorophore [23,26] whose emission properties are highly sensitive to immediate environment, although the fluorescence of the core fluorophore (aza-BODIPY) was reported insensitive to solvent [27]. Water is the most polar solvent, with a polarity index (PI) of 62.8 in terms of E_T_(30) [28]. The fluorescence of ADP(CA)_2_ in water was barely observed as shown as the first red-square point in Figure 2A. As the PI of the solvent decreases, ADP(CA)_2_ fluoresces significantly stronger. For instance, the fluorescence intensity increases by ~20 times when the solvent was changed from water to dimethyl sulfoxide (DMSO, PI = 45.1). Finally, the fluorescence intensity ratio of I_toluene_/I_water_ can be ~275 at the emission wavelength of >711 nm when excited at 655 nm. Since a very low concentration of ADP(CA)_2_ was employed (8.6 nM), we excluded the possibility that fluorescence intensity differs due to the solubility of ADP(CA)_2_. The non-radioactive decay was favored in polar solvent and competed strongly with fluorescence, so when the microenvironment was changed to non-polar solvent, ADP(CA)_2_ fluoresces strongly and exhibited a “switch-like” fluorescence emission if the vertical axis of the Figure 2A was displayed in a linear scale. The mechanism of this fluorescence “switch” is likely related to an internal photo-induced electron transfer (PeT) from the benzene moieties to the aza-BODIPY core. Such PeT resulted in fluorescence quenching that occurred more easily in polar media than non-polar media [29]. It is evidence that the fluorescence lifetime of ADP(CA)_2_ increases from ~2.3 to ~3 ns when the solvent polarity decreases. As a comparison, the data for Compound (**1**) in Figure 1 are also shown in Figure 2A. A similar conclusion can be drawn for this compound. In addition, we found that the emission peaks of ADP(CA)_2_ varied in different solvents (excited at 638 nm), such as 696, 742 and 709 nm in water, dimethyl sulfoxide (DMSO) and toluene, respectively. The quantum yields varied in different solvents (measured using Horiba Quantum-V (Horiba Scientific, Edison, USA), excited and detected at 678 and 720 nm, respectively), such as 0.56%, 7.45%, 10.51%, 12.4%, and 14.51% in water, dimethyl sulfoxide (DMSO), dioxane, dichloroethane and toluene, respectively, and 7.93% and 16.22% in ethylene glycol and glycerol, respectively.

Another possibility is that the fluorescence of ADP(CA)_2_ was significantly quenched in water due to the existence of hydrogen bonding with water [15,30]. We investigated the fluorescence intensity change as function of the water content in the mixture of water and ethylene glycol (EG, Figure 2B). EG is a highly polar solvent with a polarity index close to water. Adding a small amount of water (such as < 3% *v*/*v*) into EG does not change the polarity. Therefore, the change in fluorescence intensity of ADP(CA)_2_ in EG should not be caused by polarity. Instead, it may be due to hydrogen bonding that quenches the fluorescence. This hypothesis was verified by our results in Figure 2B. Significant decrease of fluorescence intensity was observed for both ADP(CA)_2_ and the Compound (**1**) samples. Furthermore, the fluorescence of Compound (**1**) was found extremely sensitive to water (addition of 0.033% v/v water leads to a 40% drop in intensity). This huge decrease suggests that the fluorescence of Compound (**1**) can be easily quenched by the formation of hydrogen bonding. 

#### 2.1.2. Fluorescence Response to Viscosity

**Figure 3:**
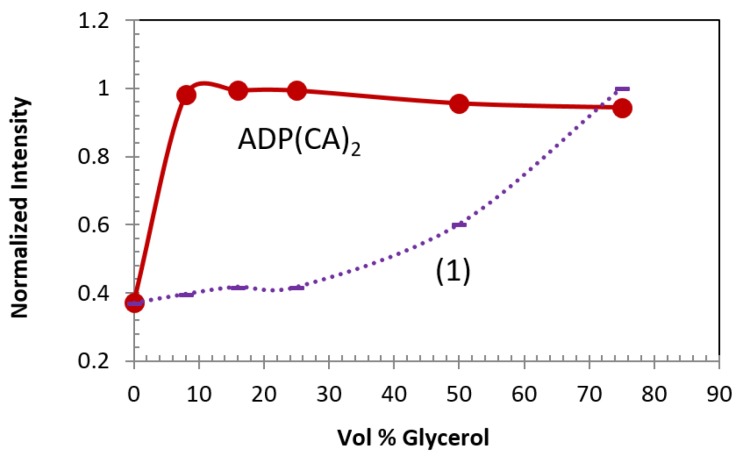
Viscosity-dependent fluorescence intensity of ADP(CA)_2_ in a glycerol/ethylene glycol mixture with different compositions. Excitation: 655 nm; Emission filter: 711/25 band-pass; Laser energy: 50 pJ (ADP(CA)_2_) and 140 pJ (Compound (**1**)).

Similar to polarity, ADP(CA)_2_’s fluorescence is also very sensitive to viscosity of the media and shows a switch-like performance (red solid line in Figure 3). We herein chose the mixture of EG (with viscosity as low as 0.0161 Pa·s [31]) and glycerol (with viscosity as high as 1.412 Pa·s [32]), because their polarity was close to each other. As shown in Figure 3, a 2.5-fold fluorescence intensity increase was observed when the volume percentage of glycerol was 8% or above (i.e., the viscosity rose). By contrast, the fluorescence of Compound (**1**) was less sensitive to the increase of viscosity, as shown as the purple dashed line in Figure 3.

#### 2.1.3. Fluorescence Response to Biological Macromolecules and Surfactant

**Figure 4:**
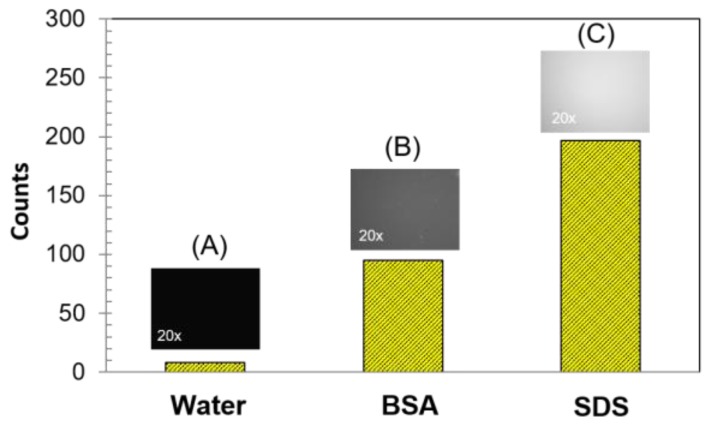
Fluorescence intensity of ADP(CA)_2_ in: water (**A**); 1% BSA solution (**B**); and 1‰ SDS solution (**C**). Excitation filter: 632/22 band-pass filter; Emission filter: 711/25 band-pass filter; Light source energy: 25%; Objective: 20×; Exposure time: 3 s.

We also investigated ADP(CA)_2_’s fluorescence change in the absence and presence of biological macromolecules. Bovine serum albumin (BSA) was selected as one example and added into the dye solution for incubation. The fluorescence of ADP(CA)_2_ in water (no BSA) was very weak (Figure 4A). The fluorescence in BSA-incubated sample was increased by 12 folds (Figure 4B), in comparison with that in the absence of BSA (Figure 4A). The hydrophobic surface on BSA favors the fluorescence enhancement, as like that in non-polar media. The polarity of BSA surface is similar with that of acetone [29] (PI = 5.1). The increase in fluorescence verified that the microenvironment surrounding ADP(CA)_2_ was changed, e.g., the polarity becomes smaller, which comes from the interaction between ADP(CA)_2_ and BSA. Besides, we cannot exclude the possibility that ADP(CA)_2_ binds to BSA so that the rotation was restricted.

Once ADP(CA)_2_ was incubated with sodium dodecyl sulfate (SDS, a type of popular surfactant molecules), its fluorescence was increased by 24 folds (Figure 4C) in comparison with that in water (Figure 4A). This enhancement suggests that some ADP(CA)_2_ may be encapsulated inside the micelle formed by SDS aggregation. The polarity of SDS micelle core was reported to be close to that of methanol in terms of E_T_(30) value [28,33]. Apparently the increase in polarity leads to the fluorescence enhancement of ADP(CA)_2_.

Taken together, we confirmed that ADP(CA)_2_’s fluorescence is highly sensitive to polarity and it can be switched on while binding to BSA or being encapsulated in micelle. This property allows us to use it as a switchable fluorescence probe for the investigation of the conformation change of protein or the protein penetration in cellular membrane in future. 

Thus far, the mechanism of the switching property of ADP(CA)_2_ is clear. It can be switched on by physical stimulation (including polarity and viscosity) and biological stimulation (the interactions with proteins). It cannot be switched by chemical simulation (including: pH and ion concentration in physiological range, as shown in our previous publication [19]).

### 2.2. Applications of ADP(CA)_2_ as a Switchable Fluorescent Probe (SFP) 

#### 2.2.1. Wash-Free Live-Cell Imaging 

**Figure 5:**
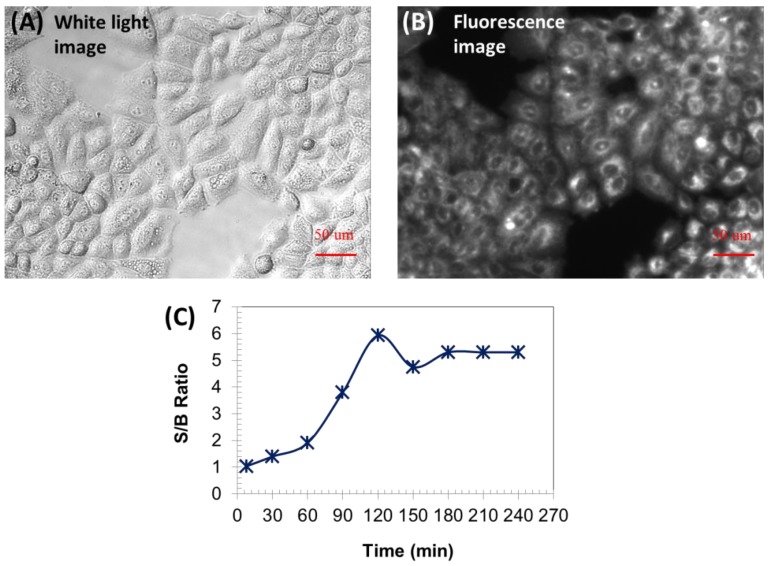
White light (**A**); and fluorescence image (**B**) of living A549 cells loaded with ADP(CA)_2_ for 120 min without washing; Excitation filter: 632/22 band-pass filter; Emission filter: 711/25 band-pass filter; Light source energy: 25%; Objective: 20×; Exposure time: 3s. (**C**) Signal to background (S/B) ratio as a function of loading time.

Wash-free live-cell imaging was carried out using adenocarcinomic human alveolar basal epithelial cells (A549 cells). The cells had been incubated with ADP(CA)_2_ (21.5 µM) for 4 h. Then the cells were imaged using a fluorescence microscope without any washing steps or removing dye-containing cell culture medium. The white light and fluorescence images are shown in Figure 5A,B respectively. As shown in Figure 5B, the cytoplasm was bright and showed well-distributed fluorescence while the nucleus stayed dark (no fluorescence). Since cytoplasm contains abundant membrane structures (e.g., endoplasmic reticulum) and all kinds of proteins, ADP(CA)_2_ would have interactions with them so that the fluorescence of ADP(CA)_2_ can be switched on significantly. Furthermore, the intracellular viscosity is higher and inhomogeneous, especially the perinuclear region is of the largest viscosity [20]. Thus, we believed that the fluorescence of ADP(CA)_2_ inside the cells is a comprehensive enhancement by biological and physical stimulation. 

The dye-containing cell culture medium showed very weak fluorescence compared to cells (Figure 5B). Under the given conditions, the contrast, i.e., signal-to-background (S/B) ratio between intracellular fluorescence signal and extracellular background, can achieve 6 after 120 min incubation (Figure 5C). We continued measuring the contrast for two days, and it remained at 5.3. The high contrast and longtime intracellular fluorescence of ADP(CA)_2_ make it an excellent fluorescent probe for wash-free live-cell imaging and in vivo cell tracking in future. Note that we also imaged the cells before incubating them with the dye solution (i.e., no ADP(CA)_2_). We found that the image was very dark in the entire field of view, which meant that the laser leakage was ignorable compared with the background fluorescence signal. 

The cellular uptake will become more efficient if the fluorophore has hydrophilic moieties [21]. We conjugated ADP(CA)_2_ with a water-soluble and temperature sensitive polymer and used the conjugate for wash-free cell imaging. The three components of the polymer are *N*-isopropylacrylamide (NIPAM), *N*-tert-butylacrylamide (TBAm) and allylamine (AH) with a molar ratio of 185:15:1 (i.e., one of the three polymers in Figure 7 and the details can be found in Methods). Under the same incubation time (e.g., 120 min), the brightness of cytoplasm was found much larger than that using ADP(CA)_2_ alone (Table 1). This is likely attributed to a faster cellular uptake towards ADP(CA)_2_~Polymer conjugate. Without sacrificing the contrast much, the high brightness permits the use of much lower light source, which in turn will be beneficial to the prevention from photo-bleaching during imaging process. 

#### 2.2.2. Fluorescence Imaging in Live Animals 

**Figure 6:**
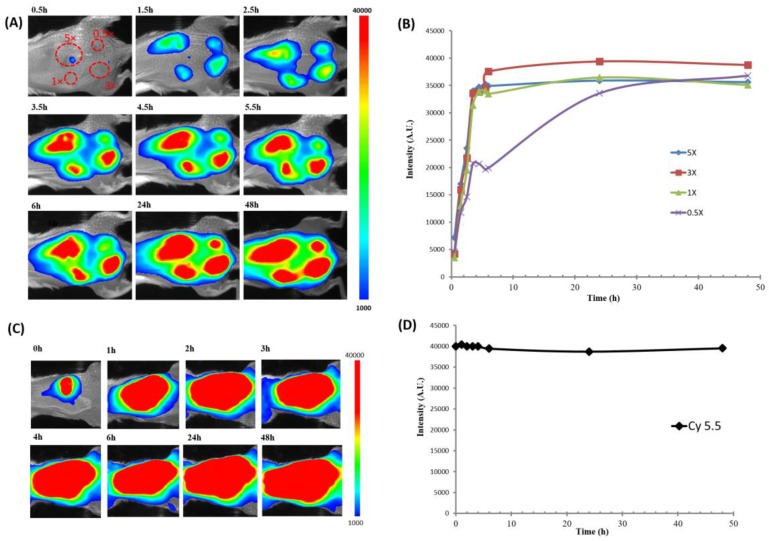
ADP(CA)_2_ solutions with four different concentrations or Cy5.5 solution were injected subcutaneously in the back of animals. (**A**) In vivo fluorescence images of ADP(CA)_2_ at different time points; (**B**) The mean fluorescence intensities of ADP(CA)_2_ at the four injection sites as a function of time; (**C**) In vivo fluorescence images of Cy5.5 at different time points; (**D**) The mean fluorescence intensity of Cy5.5 as a function of time; Those fluorescence images were taken using KODAK In Vivo FX Pro system (f-stop: 2.5, excitation filter: 630 nm, emission filter: 700 nm, 4 × 4 binning; Carestream Health, Rochester, NY, USA). All data analyses were performed using Carestream Molecular Imaging Software, Network Edition 4.5 (Carestream Health). A.U.: arbitrary units.

In order to further demonstrate the switching property of ADP(CA)_2_ in in vivo fluorescence imaging, ADP(CA)_2_ solutions with four different concentrations were injected subcutaneously in the back of a mouse. As shown in the first image in Figure 6A, four injecting sites were circled in red color corresponding to four concentrations (0.5×: 10.75 μM; 1×: 21.5 μM; 3×: 64.5 μM; and 5×: 107.5 μM). After injection, whole body fluorescence images were recorded at different time points (Figure 6A). The results showed that ADP(CA)_2_ did not fluoresce immediately after the injections. A substantial increase in fluorescence intensity was found at the injection sites after 30 min (Figure 6A,B). That is because only the dye that entered the cells can be switched on and the cellular uptake process takes time. With the time increase, more dye molecules were uptaken into the cells. Therefore, the fluorescence intensity increased at the four sites as a function of time. After 5 h, the fluorescence intensity increased 9.7 times at 1× site which is shown as green line in Figure 6B. The final fluorescence intensity depends on how much probe the cells can uptake. Based on our results, 21.5 μM is the best concentration considering the fluorophore dose and fluorescence signal intensity. We also found out the cellular uptake speed is independent of the probe concentration. As a control, another common NIR fluorophore Cy5.5 with a concentration of 21.5 μM was injected subcutaneously in the back of a mouse following the same manner. The mean intensity of Cy5.5 as a function of time after injection is shown in Figure 6D. The results clearly showed that this fluorophore does not have a unique switching property. It was consistently bright from the beginning of the injection until the end point of detection. The in vivo results further confirmed the previous conclusion. We think that when ADP(CA)_2_ working as the SFP in wash-free live-cell imaging and in vivo tissue fluorescence imaging, only intracellular microenvironment could turn on its fluorescence and the fluorescence can be remained for at least two days.

#### 2.2.3. Temperature Sensing 

**Figure 7:**
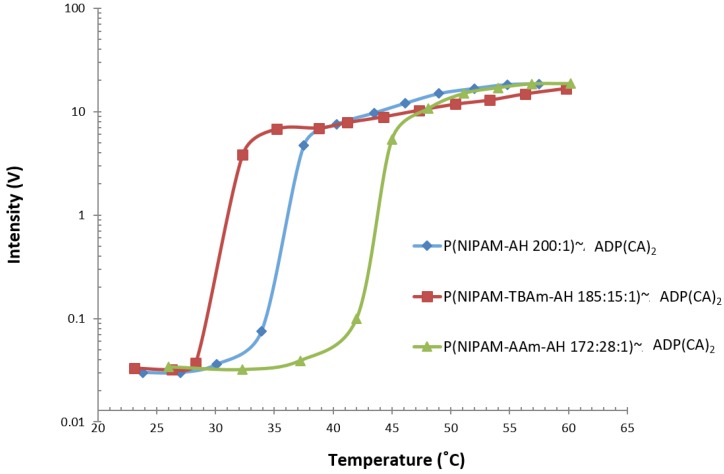
Fluorescence intensity changes of ADP(CA)_2_-conjugated thermosensitive polymers as a function of temperature; The three temperature probes are P(NIPAM-AH 200:1)~ADP(CA)_2_, P(NIPAM-TBAm-AH 185:15:1)~ADP(CA)_2_ and P(NIPAM-AAm-AH 172:28:1); Excitation wavelength: 655 nm; Emission filter: 711/25 band-pass filter and a neutral density filter with OD 0.9. Laser energy: 700pJ. OD: optical density.

Besides directly using ADP(CA)_2_ as the SFP, ADP(CA)_2_-conjugated polymers were also developed as the SFPs for temperature sensing. Based on our previous work [16,17], thermosensitive polymer of poly(*N*-isopropylacrylamide) (PNIPAM) was found that it could efficiently change the microenvironment for the environment-sensitive fluorophores when responding to temperature. When the temperature crosses the LCST of the thermo-sensitive polymers, these polymers experience a reversible phase transition. This phase transition leads to a significant change in polarity and viscosity microenvironment. Since ADP(CA)_2_ was found extremely sensitive to its microenvironment (especially polarity and viscosity), we expect that the fluorescence emission from these ADP(CA)_2_-conjugated polymers would be sensitive to the temperature. Different ADP(CA)_2_-conjugated thermo-sensitive polymers were synthesized: (1) P(NIPAM-AH 200:1)~ADP(CA)_2_; (2) P(NIPAM-TBAm-AH 185:15:1)~ADP(CA)_2_; and (3) P(NIPAM-AAm-AH 172:28:1)~ADP(CA)_2_ (AAm: acrylamide). The detailed protocol has been discussed in our previous paper [16]. The relationship between the fluorescence strength of these probes and temperature has been investigated and shown in Figure 7. The results indicate these probes have three different LCSTs, 28.3, 34 and 42 °C. The fluorescence of these probes was at “OFF” state when the temperature is below LCST. Once the temperature is across the LCST, the fluorescence was significantly turned on. The temperature sensing range was found to be 28.3–32.8 °C, 34–38 °C and 42–46 °C (Table 2). Within these ranges, the fluorescence ratios between ON and OFF states (I_ON_/I_OFF_) were found to be 304-fold, 318-fold and 284-fold for the three probes, respectively. The average temperature sensitivity was 73-fold per Celsius degree. To the best of our knowledge, this is the highest sensitivity. In addition, the LCST can be adjusted by introducing hydrophobic (i.e., TBAm) or hydrophilic (i.e., AAm) monomers with different ratios so that more temperature ranges can be covered in the future.

#### 2.2.4. Ultrasound-Switchable Fluorescence (USF) Imaging in Tissue-Mimicking Phantoms 

**Figure 8:**
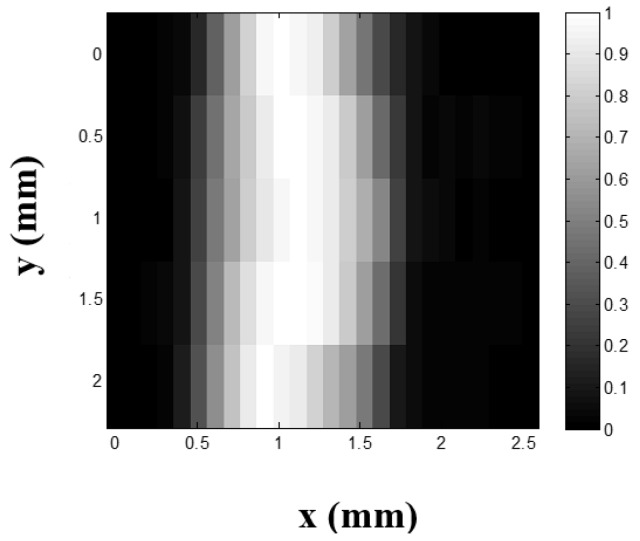
The ultrasound-switchable fluorescence (USF) image of the micro-tube embedded into the tissue-mimicking phantom; ADP(CA)_2_-encapsulated thermo-sensitive nanocapsules were used as the contrast agents.

USF imaging has been proposed recently by our group to achieve high-resolution fluorescence imaging in centimeter-deep tissue with high SNR and sensitivity [17,19,22]. The principle of USF imaging is based on the unique switching properties of the contrast agent whose fluorescence can be switched on and off via a focused ultrasound wave. In this case, an excellent contrast agent (SFP) for USF imaging based on ADP(CA)_2_ was developed by encapsulating ADP(CA)_2_ fluorophores into thermosensitive nanocapsules [19]. The switching principle is similar to temperature SFP we developed in Section 2.2.3. To show the performance of this type of SFP in deep-tissue high-resolution imaging, USF imaging was performed in tissue-mimicking silicone phantoms with a thickness of 11 mm. Figure 8 shows the USF image of a micro-tube embedded in the tissue-mimicking phantom on x-y plane (top view). The tube size is 310 µm inner diameter (I.D.) and 640 µm outer diameter (O.D.). The formation of the phantom sample can be found in the Methods. In this study, the micro-tube was filled with aqueous solution of ADP(CA)_2_-encapsulated nanocapsules. The full-width-at-half-maximum (FWHM) of the USF image profile along x direction at each y location was calculated. The average FWHM at different y locations was 0.93 ± 0.07 mm, which is slightly larger than the outer diameter of tube (O.D.: 0.64 mm). Our results indicate that the micro-tube was successfully imaged in centimeter-deep tissue by USF with high resolution (~900 µm) and high signal-to-background contrast (~79).

## 3. Conclusions

We have investigated the switching mechanism and multiple applications of a recent NIR aza-BODIPY fluorophore, namely ADP(CA)_2_. Our results indicate that ADP(CA)_2_ is an extremely environment-sensitive dye, showing very weak fluorescence in aqueous solution whereas strong fluorescence in non-polar and high-viscosity media. Because of the unique switching property, ADP(CA)_2_ has been successfully applied in wash-free live-cell imaging, fluorescence imaging in live animals, temperature sensing, and USF imaging. More applications of this probe will be discovered in the future. 

## 4. Materials and Methods

### 4.1. Conjugates of ADP(CA)_2_ and NIPAM Polymer

*N*-isopropylacrylamide (NIPAM), *N*-tert-butylacrylamide (TBAm), acrylamide (AAm), allylamine (AH), *N*,*N*,*N*’,*N*’-tetramethyl ethylene diamine (TEMED), ammonium persulfate (APS), *N*-(3-dimethylaminopropyl)-*N*’-ethylcarbodiimide hydrochloride (EDC) and fetal bovine serum (FBS) were purchased from Sigma-Aldrich (St. Louis, MO, USA) and used as received. RPMI Medium 1640 powder and Pen Strep were purchased from Life Technologies (Grand Island, NY, USA). The polymer synthesis and conjugation protocol were adopted from our previous work [16].

### 4.2. Fluorescence Measurement of ADP(CA)_2_ under Varying Micro-Environment Conditions

The fluorescence measurement system was adopted according to our previous report [16]. Briefly, the excitation wavelength was changed into 655 nm and a 711/25 band-pass filter was adopted as the emission filter to reject excitation photons. No temperature control accessories were applied in the current section. 

A stock solution was prepared by dissolving ADP(CA)_2_ in methanol at a concentration of 20 mg/mL (21.5 mM). 

#### 4.2.1. Polarity

Five solvents with different polarity indexes [28] were selected: water (62.8), dimethyl sulfoxide (DMSO, 45.1), 1,2-dichloroethane (41.3), 1,4-dioxane (36), and toluene (33.9). The final concentration of ADP(CA)_2_ solution was set as 8.6 nM for measurement, with a total volume of 3 mL.

#### 4.2.2. Viscosity

Eight solutions with different viscosities were prepared by mixing glycerol and ethylene glycol at different volume ratios, including 0/100, 8/92, 16/84, 25/75, 50/50, 75/25, 90/10 and 100/0 of glycerol/ethylene glycol. The final concentration of ADP(CA)_2_ solution was set as 8.6 nM, with a total volume of 3 mL.

#### 4.2.3. Interaction with Biological Macromolecules and Surfactant

Bovine serum albumin (BSA) solution (1%, *w*/*v*) and sodium dodecyl sulfate (SDS) solution (1%, *w*/*v*) were prepared. The final concentration of ADP(CA)_2_ solution for measurement was 21.5 µM with a total volume of 0.5 mL. The fluorescence measurement was performed based on a cell imaging system described below. The ADP(CA)_2_ was excited at ~632 nm using a lamp and a band-pass filter (632/22 nm band pass, central wavelength: 632 nm; bandwidth: 22 nm). The emission filter was 711/25 band pass filter (central wavelength: 711 nm; bandwidth: 25nm).

### 4.3. Wash-free live-cell Imaging 

The cell imaging system consists of an inverted fluorescence microscope (Nikon Instruments Inc., Model: Eclipse Ti-U, Melville, NY, USA) and a high-resolution CCD camera (ANDOR, Model: DR-328G-C01-SIL, South Windsor, CT, USA). 

Adenocarcinomic human alveolar basal epithelial cells (A549, ATCC, Manassas, VA, USA) were cultured at 37 °C using RPMI medium supplemented with 10% FBS and 100 U penicillin–streptomycin (Pen-Strep) for use. Prior to imaging, A549 cells were seeded in a 48-well plate with a density of 12,000 cells/cm^2^. After 24 h, cell loading was carried out. For pure ADP(CA)_2_ cell imaging, 10.75 nmol ADP(CA)_2_ in methanol was added into 500 µL cell media (per well). For ADP(CA)_2_~Polymer cell imaging, 7 nmol ADP(CA)_2_~Polymer was added into 500 µL cell media (per well). The water-soluble polymer was P(NIPAM-TBAm-AH 185:15:1). The fluorescence images were taken directly at the indicated time points without either washing or removing ADP(CA)_2_-containing culture media. The ADP(CA)_2_ was excited at ~632 nm using a lamp and a band-pass filter (632/22 nm band pass, central wavelength: 632 nm; bandwidth: 22 nm). The emission filter was 711/25 band pass filter (central wavelength: 711 nm; bandwidth: 25 nm). The microscope settings were kept the same in all imaging experiments (objective: 20×; exposure time: 3 s). The laser intensity was optimized depending on different imaging experiments.

### 4.4. Fluorescence Imaging in Live Animals

Female Balb/c mice (20–25 g), purchased from Taconic Farms (Germantown, NY, USA), were used in this study. The animal protocols were approved by the University of Texas at Arlington’s Animal Care and Use Committee. To perform fluorescence imaging in live mice, ADP(CA)_2_ solutions (100 μL) with four different concentrations (10.75 μM, 21.5 μM, 64.5 μM and 107.5 μM) or Cy5.5 solution (50 μL) with a concentration of 21.5 μM were injected subcutaneously in the back of the mice. After injection, whole body fluorescence images were taken subsequently at various time points. Those fluorescence images were taken using KODAK In Vivo FX Pro system (f-stop: 2.5, excitation filter: 630 nm, emission filter: 700 nm, 4 × 4 binning; Carestream Health, Rochester, NY, USA). After background correction, regions of interest were drawn over the implantation locations in the fluorescence images and the mean fluorescence intensities for all pixels in the regions of interest were calculated. All data analyses were performed by using Carestream Molecular Imaging Software, Network Edition 4.5 (Carestream Health).

### 4.5. Temperature Sensing

The fluorescence measurement system was adopted according to our previous report [16]. In this study, the excitation wavelength was selected as 655 nm. A 711/25 band-pass filter and a neutral density filter with O.D. 0.9 was adopted as the emission detection filter. 

### 4.6. Ultrasound-Switchable Fluorescence (USF) Imaging in Tissue-Mimicking Phantoms

Tissue-mimicking silicone phantoms: the silicone kit was purchased from Factor II Inc. (VST-50: VerSilTal Silicone Elastomer; Lakeside, AZ, USA). The kit includes two major components: silicone elastomer and catalyst. We constructed the tissue-mimicking phantom using silicone (to mimic tissue’s acoustic properties) doped with TiO_2_ (to mimic tissue’s optical scattering properties). The estimated absorption coefficient µ_a_ = 0.03 cm^−1^ and reduced scattering coefficient µ_s_’ = 3.5 cm^−1^. The mixture solution was poured into a plastic container and a silicone micro-tube was inserted in the middle. The container was then placed into a vacuum to remove air bubbles inside the mixture for 20 min. After 6 h, the tissue-mimicking silicone phantom was ready to use. 

The USF imaging system was adopted from our recent paper [19]. In this work, a small silicone tube with inner diameter of 310 µm and outer diameter of 640 µm was filled with the aqueous solution of the ADP(CA)_2_ contrast agents and embedded into the tissue-mimicking phantom to simulate a blood vessel as the target for USF imaging. The thickness of the tissue phantom is ~11 mm. The excitation light source was a diode laser (MLL-FN-671, Changchun New Industries Optoelectronics Technology Co. Ltd., Changchun, China). One 650/60 band-pass filter (central wavelength: 650 nm; bandwidth: 60 nm) was applied as the excitation filter and two long-pass filters (edge wavelength: 715 nm) were adopted as the emission filter. A rectangular area was scanned by the high-intensity-focused-ultrasound (HIFU) transducer.

## Figures and Tables

**Table 1:** The comparison between ADP(CA)_2_ and ADP(CA)_2_~Polymer conjugate.

Probe	S/B Ratio	Fluorescence Intensity Counts	Loading Time
ADP(CA)_2_	5.93	172 (light source: 50%)	120 min
ADP(CA)_2_~Polymer	3.75	255 *(light source: 50%)	120 min
ADP(CA)_2_~Polymer	4.5	143 (light source: 25%)	120 min

* The intensity is saturated. S/B ratio: signal to background.

**Table 2:** Overview of temperature probes.

Probe	λ_ex_,λ_em_ (nm)	I_On_/I_off_	T_th_ (°C)	T_BW_ (°C)
P(NIPAM-AH 200:1)~ADP(CA)_2_	655 & 711	304	34	4
P(NIPAM-TBAm-AH 185:15:1)~ADP(CA)_2_	655 & 711	318	28.3	4.5
P(NIPAM-AAm-AH 172:28:1)~ADP(CA)_2_	655 & 711	284	42	4

**T_th_**: temperature threshold (LCST, lower critical solution temperature); **T_BW_**: transition bandwidth (temperature sensitive range).
